# Sunagoke Moss (*Racomitrium japonicum*) Used for Greening Roofs Is Severely Damaged by *Sclerotium delphinii* and Protected by a Putative *Bacillus amyloliquefaciens* Isolate

**DOI:** 10.3389/fmicb.2019.00372

**Published:** 2019-02-28

**Authors:** Mako Tamura, Minatsu Tanabe, Jari P. T. Valkonen, Motomu Akita

**Affiliations:** ^1^Department of Biotechnological Science, Graduate School of Biology-Oriented Science and Technology, Kindai University, Wakayama, Japan; ^2^Department of Agricultural Sciences, University of Helsinki, Helsinki, Finland

**Keywords:** bryophyte, pathogen, antagonist, *Racomitrium japonicum*, *Sclerotium delphinii*, *Bacillus amyloliquefaciens*

## Abstract

Mosses are ecologically important plants also used for greening, gardening, and decorative purposes. Knowledge of the microbial flora associated with mosses is expected to be important for control and preservation of global and local environments. However, the moss-associated microbial flora is often poorly known. Moss-associated fungi and bacteria may promote plant growth and pest control, but they may be alternative hosts for pathogens of vascular plants. In this study, the fungus *Sclerotinia delphinii* was identified for the first time as a pathogen that causes severe damage to Sunagoke moss (*Racomitrium japonicum*). This moss is used for greening roofs and walls of buildings in urban environments owing to its notable tolerance of environmental stresses. Inoculation with the *S. delphinii* strain SR1 of the mono- and dicotyledonous seed plants *Hordeum vulgare*, *Brassica rapa* var. *pekinensis*, *Lactuca sativa*, and *Spinacia oleracea*, in addition to the liverwort *Marchantia polymorpha* and the moss *Physcomitrella patens*, showed that the fungus has a wide host range. Colonization with SR1 progressed more rapidly in non-vascular than in vascular plant species. Studies with *P. patens* under controlled conditions showed that SR1 secreted a fluid during colonization. Treatment with the secretion induced production of reactive oxygen species in the moss. Endogenous peroxidase partially inhibited SR1 colonization of *P. patens*. A bacterial isolate, most likely *Bacillus amyloliquefaciens*, that coexists with *R. japonicum* was antagonistic to SR1 growth. Taken together, the present results suggest that fungal colonization of mosses may be prevented by a peroxidase secreted by the moss and an antagonistic bacterium coexisting in the moss habitat. The findings suggest that there is potential to apply biological control measures for protection of mosses against fungal pathogens.

## Introduction

Sunagoke moss (*Racomitrium japonicum* Dozy & Molk.; Grimmiaceae) has been used for greening of roofs and walls of buildings in an urban environment (Supplementary Figure S1). Given the notable tolerance of this species to environmental stresses, such as high temperature, drought, and nutrient limitation, plants can be maintained without irrigation and fertilization for several years. The use of Sunagoke moss for greening has been studied previously (Ushada et al., 2007; Hendrawan and Murase, 2011). The most important trait that promotes selection of this moss as material for covering buildings is its impressive “light moss-green” color when the colony is maintained under appropriate conditions. Therefore, it is important to establish techniques to maintain the moss colony in good health for as long as possible. Moss health and quality can be impaired by inadequate management, excessive watering, and/or attack by plant pathogens. In particular, this moss is occasionally attacked by pathogenic fungi, which cause severe deterioration in moss quality. In the present study, we describe a devastating disease of Sunagoke moss caused by a fungus and discuss possibilities to control the disease.

Bryophytes provide an important habitat for diverse groups of microorganisms. Therefore, moss–microbe interactions have been studied from ecological and biological perspectives (Hoshino et al., 2001; Davey and Currah, 2006; Davey et al., 2009a,b, 2010; Huhtinen et al., 2010; Stenroos et al., 2010). We previously highlighted that *R. japonicum* colonies used for greening can be attacked by several fungi (Akita et al., 2011), including pathogens of seed plants (Lehtonen et al., 2012b). Hence, mosses may be alternative hosts of fungi pathogenic to seed plants, which justifies further study from the point-of-view of pest control in agriculture. In addition, from a physiological perspective, mosses have been suggested to be good model systems for the study of plant responses during pathogenesis, because they may have pathogen recognition and signal transduction systems similar to those of vascular plants. Several pathogens of vascular plants, such as the bacteria *Erwinia* spp. (Andersson et al., 2005; de Leon et al., 2007; de Leon and Montesano, 2013; Machado et al., 2015; Alvarez et al., 2016) and fungi belonging to the oomycete *Pythium* (Oliver et al., 2009; Takikawa et al., 2015; Castro et al., 2016) and *Botrytis* (de Leon et al., 2012; Castro et al., 2016), cause disease symptoms and expression of disease-associated genes that encode pathogenesis-related proteins when artificially inoculated to the moss *P. patens* (Hedw.) Bruch & Schimp. (Castro et al., 2016). Involvement of signal transduction through the MAPK cascade has been demonstrated following application of chitosan, an elicitor of pathogen defense responses, to *P. patens* (Bressendorff et al., 2016).

**FIGURE 1:**
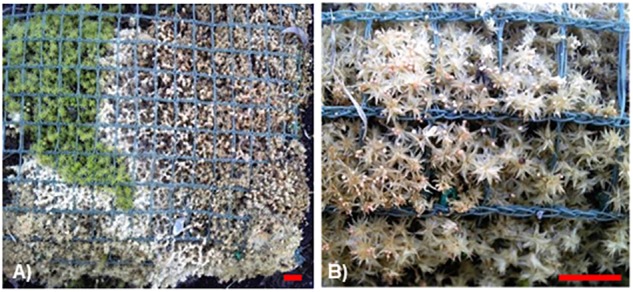
Infected and damaged *Racomitrium japonicum* colony in a panel used for roof greening. **(A)** Bleached lesion caused by fungal colonization. **(B)** Sclerotia formation on the damaged lesion. Blue-green nets with grid size of 1 cm are used for fixing the moss plants in place. Red scale bars = 1 cm.

In our previous study, the majority of pathogenic fungi isolated from *R. japonicum* (Akita et al., 2011) were considered to be opportunistic pathogens that usually cause relatively mild symptoms. However, more recently, we detected a fungus that causes development of devastating symptoms in the moss colonies (Figure 1A). Infected plants are bleached and the fungus produces sclerotia (Figure 1B). In the present study, we observed the pathogenesis of this fungus, determined as *Sclerotium delphinii* Welch, on several mosses, including *P. patens*, the liverwort *M. polymorpha* L., and seed plants. It is the first report that *S. delphinii* can infect bryophytes and liverworts. *Sclerotium* species include oxalate-secreting and reactive oxygen species (ROS) generating fungi for which plant oxalate metabolism may be involved in symptom development (Kim et al., 2008; Williams et al., 2011). In the current study, we demonstrate that ROS can be induced in a moss by fungal secretion. We show the involvement of peroxidase in partial inhibition of fungal colonization and propose a possible approach to protect mosses from fungal pathogens by application of a moss-associated bacterium. Given that moss habitats are promising sources of microorganisms beneficial to vascular plants, such as antagonists of pathogenic fungi (Shcherbakov et al., 2013), the present results indicate the importance of maintaining habitats of mosses and the associated beneficial microorganisms.

## Materials and Methods

### Isolation and Culture of Fungi

Fungal sclerotia (ca. 2 mm diameter, see Figure 1B) were collected from a symptomatic colony of *R. japonicum* and sterilized with sodium hypochlorite solution (8.5 to 13.5% active chlorine; Nakalai Tesque, Kyoto, Japan) diluted 10-fold [0.05% (w/w) Tween 20] for 2 min, followed by five rinses with sterilized water. Sclerotia were cultured on potato dextrose agar (PDA) in Petri dishes under continuous light-emitting diode (LED) illumination (mixture of white and red) of ca. 80 μmol m^-2^ s^-1^ at 23°C. The hyphae that developed from a sclerotium were selected and subcultured to establish a single fungal isolate, SR1, used for further study. The fungus was maintained on PDA medium (Nissui Pharmaceutical, Tokyo, Japan) or potato sucrose agar (PSA) containing 3.9 g L^-1^ potato extract (Formedium, Hunstanton, United Kingdom), 21 g L^-1^ sucrose and 14.1 g L^-1^ agar under continuous light and the conditions described above.

### Plant Materials

In our previous study, the peroxidase-encoding gene *Prx34* was knocked out in *P. patens* (Lehtonen et al., 2009). The wild type and a *Prx34*-deficient strain of *P. patens* (Prx34-KO) were aseptically used. An aseptic culture of *M. polymorpha* was kindly provided by Dr. K. T. Yamato, Kindai University, Wakayama, Japan. Gametophytes of the mosses *R. japonicum* and *Hypnum plumaeforme* Wilson were collected from the campus of the Faculty of Biology-Oriented Science and Technology, Kindai University, Wakayama, Japan. The collected gametophytes were washed in tap water for 60 min prior to use. Seeds of cultivated dicotyledonous plants, comprising *Hordeum vulgare* L., *Brassica rapa* L. var. *pekinensis* (Lour.) Kitam., *L. sativa* L., and *S. oleracea* L., were purchased from a local market and surface-sterilized with sodium hypochlorite for 10 min, followed by three rinses with sterilized water. The seeds were germinated in autoclaved Plant Boxes (60 mm × 60 mm × 100 mm; AGC Techno Glass, Shizuoka, Japan) filled with vermiculite (depth 3 cm) (size 3–6 mm, Komeri, Niigata) and 55 mL of commercial liquid fertilizer (Hyponex 6-10-5, Hyponex Japan, Osaka, Japan) diluted 1000-fold. Plants were cultured under continuous LED illumination (mixture of white and red) of ca. 80 μmol m^-2^ s^-1^ at 23°C.

### Culture Conditions for *P. patens*

Protonemata of *P. patens* were cultured on BCD plate medium (1 mM MgSO_4_, 1.85 mM KH_2_PO_4_, 10 mM KNO_3_, 45 mM FeSO_4_, 1 mM CaCl_2_, 0.22 mM CuSO_4_, 0.19 mM ZnSO_4_, 10 mM H_3_BO_4_, 0.10 mM Na_2_MoO_4_, 2 mM MnCl_2_, 0.23 mM CoCl_2_, and 0.17 mM KI, solidified with 8 g L^-1^ agar) supplemented with 5 mM ammonium tartrate^1^. Protonemata (ca. 1 g) were homogenized 30 s in 15 mL of sterilized soft agar (1.5 g L^-1^ in water) using a handy homogenizer (Polytron PT1200E, Kinematica, Luzern) for subculturing. A cellophane sheet (No. 300, Rengo, Osaka) was put on the medium and 3 mL of the homogenate was poured on the plate and subcultured every 2 weeks. *In vitro* gametophytes of *P. patens* were developed on BCD plate medium by culture for 3 weeks under continuous illumination from a cold cathode fluorescent lamp with ca. 80 μmol m^-2^ s^-1^ at 23°C.

### Fungus Identification

Genomic DNA was extracted from fungal hyphae grown in aseptic culture. Hyphae were frozen in liquid nitrogen, ground in a mortar, and the powder was suspended in 300 μL lysis buffer (100 mM Tris–Hc1, pH 7.5, SDS 0.5% w/v, 30 mM EDTA) and boiled for 7 min. Subsequently, 150 μL of 3 M sodium acetate was added, followed by incubation at -30°C in a freezer for 7 min. After centrifugation at 13,000 ×*g* for 5 min, the supernatant was extracted with an equal volume of phenol:chloroform:isoamyl alcohol (1:1:1, v/v/v) and washed with chloroform, 2-propanol, 70% (v/v) ethanol and then desiccated. Finally, the purified DNA was suspended in TE (10:1) buffer. The rDNA internal transcribed spacer (ITS) region, including ITS1 and ITS2, was amplified by PCR for sequencing using KOD FX Neo polymerase (TOYOBO, Osaka, Japan) in accordance with the manufacturer’s protocol. The primers NSI1 (5^′^-GATTGAATGGCTTAGTGAG-3^′^) and NLB4 (5^′^-GGATTCTCACCCTCTATGA-3^′^) (Martin and Rygiewicz, 2005) were used. PCR products were analyzed by agarose gel electrophoresis, extracted from the gel using the QIAEX^®^ II Gel Extraction Kit (QIAGEN, Velo, Netherlands) and sequenced by SigmaGenosys (Sigma-Aldrich Japan, Tokyo, Japan).

### Pathogenicity Test on Non-vascular and Vascular Plants

Pathogenicity of the isolated fungus on *R. japonicum* was tested in Petri dishes (diameter 8.5 cm, height 4 cm) containing 15 g vermiculite and 40 mL of 1000-fold diluted commercial liquid fertilizer (Hyponex 6-10-5). The Petri dishes were autoclaved, the gametophytes were transferred onto the vermiculite, then the Petri dishes were packed into a transparent plastic box (10 cm × 20 cm × 6 cm). To test the pathogenicity on non-vascular plants, gametophytes of *H. plumaeforme* and *P. patens*, and thalli of *M. polymorpha* were transferred to autoclaved Plant Boxes (60 mm × 60 mm × 100 mm) containing vermiculite (depth 3 cm) and 55 mL BCD medium. Fungal hyphae cultured for 2 weeks on PDA plates were cut into ca. 0.5 cm^2^ pieces and placed on the vermiculite at a minimum distance of 1.5 cm from the plants. All Petri dishes and Plant Boxes were incubated under continuous LED illumination of ca. 80 μmol m^-2^ s^-1^ at 23°C.

### Pathogenicity Tests on *P. patens*

To test the pathogenicity of the fungal isolate against the protonema of *P. patens*, a sclerotium was placed directly on protonemata that had been cultured on BCD medium in a 9 cm Petri dish for about 1 week. To test pathogenicity against the gametophyte of the WT and Prx-KO mutant of *P. patens*, explants were collected from colonies cultured on BCD medium for 3 weeks. Eight gametophytes per plate were concentrically inoculated, each 1.5 cm from the center of the plate, on BCD medium in a 9 cm Petri dish and the sclerotium was placed at the center of the dish. For the pathogenicity test using the gametophyte colonies, six gametophytes were concentrically placed with each 2.0 cm apart from the center on BCD medium in a 9 cm Petri dish and cultured for 3 weeks. A sclerotium was then placed at the center of the dish. All experiments were carried out under continuous light conditions using a cold cathode fluorescent lamp with light intensity of about 80 μmol m^-2^ s^-1^ at 23°C.

### Detection of ROS

A cuvette (12 i.d. × 55 mm) was filled with 895 μL of 50 mM sodium acetate buffer (pH 5.0) and a sheet of 1 cm^2^ of the protonemata directly cut from the 2 weeks old culture plate. After incubation for 30 min in the dark at room temperature, 5 μL of 1 mM 2-methyl-6-(4-methoxyphenyl)-3,7–dihydroimidazo(1,2-a)pyrazin-3-one hydrochloride (MCLA; Sigma-Aldrich, St. Louis, United States) and secreted material (100 μL) directly collected by careful pipetting from the fungus was added. Measurement with a luminescence meter (Gene Light GL-200, Microtec, Chiba, Japan) was started immediately. The measurement time was set to 5 s.

### Simultaneous Inoculation With the Fungus and Its Antagonist

The antagonistic bacterial strain R-1 (Supplementary Figure S2) was found by chance in a study for isolating fungi coexisting with moss colonies. The 16S rDNA sequence of R-1 (NCBI accession no. AB847954) showed extremely high similarity to that of *Bacillus amyloliquefaciens* (99% identity). The bacterium was cultured in LB medium for 19 h at 28°C, collected by centrifugation at 13,000 ×*g* for 10 min, and resuspended in 10-fold diluted BCD medium at OD_600_ = 0.1. An aliquot (5 μL) of the suspension was inoculated onto gametophytes of *P. patens* cultured on BCD plates, which were cultured under continuous light (ca. 80 μmol m^-2^ s^-1^) at 23°C for 1 week. Pure-cultured sclerotia (2 mm in diameter) were set 1.5 cm from single gametophytes or 2 cm from the colony of gametophytes as described in the preceding sections.

## Results

### *Racomitrium japonicum* Is Severely Damaged by *Sclerotium delphinii*

**FIGURE 2:**
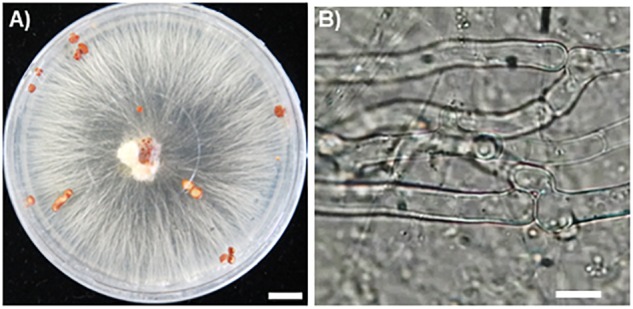
Pure culture of SR1, a pathogenic fungus isolated from *R. japonicum*. **(A)** Growth of hyphae and formation of sclerotia on PDA medium. Bar = 1 cm. Note that secretory fluid is observable at the inoculation site in the center of the plate and sclerotia are developing in different areas of the plate. **(B)** A clamp connection observed near the septum. Bar = 10 μm.

**FIGURE 3:**
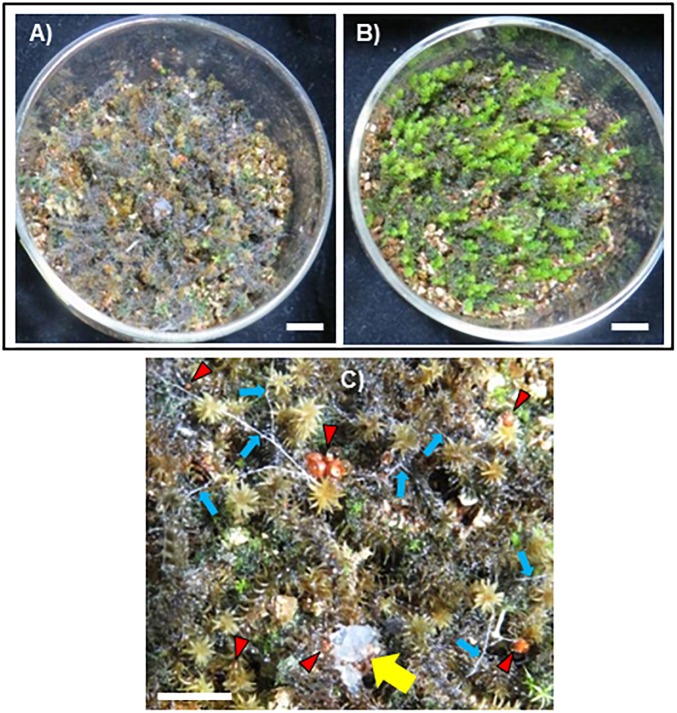
Fungal isolate SR1 is a pathogen of *R. japonicum*. **(A)** Damage to *R. japonicum* caused by colonization with the fungal isolate SR1. **(B)** Non-inoculated control. **(C)** Sclerotia of SR1 developing on inoculated *R. japonicum* (red arrowheads). The yellow arrow indicates the inoculated agar piece. Note that characteristic bundles of hyphae were formed (blue arrows). Bars = 1 cm.

Hyphae of the fungal isolate SR1 grew well on PDA and PSA plates, and white or slightly brown sclerotia formed 3 weeks after inoculation (Figure 2A). Microscopic observations showed that the hyphae had clamp connections (Figure 2B). After inoculation of *R. japonicum* with the pure-cultured hyphae, the gametophytes were severely damaged and sclerotia developed (Figure 3). This result indicated that the fungal isolate, SR1, is a destructive pathogen of *R. japonicum*. The ITS sequence (LC374375) of SR1 was 100% identical with that of *S. delphinii* strain CBS672.71 (JN241578.1), which suggested that SR1 is a strain of *S. delphinii* (syn. *S. rolfsii* var. *delphinii*).

### The Fungal Isolate SR1 Has a Wide Host Range, Including Non-vascular and Seed Plants

**FIGURE 4:**
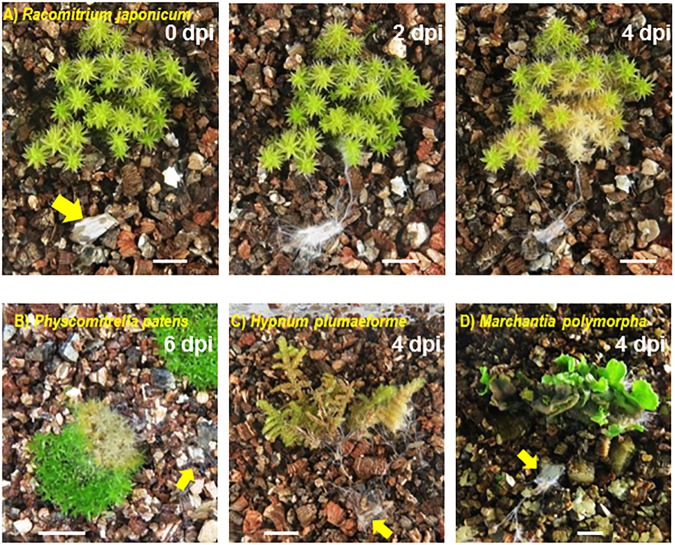
Fungal isolate SR1 is a multihost pathogen of mosses and liverworts. One piece of agar with hyphae of SR1 (yellow arrow) was placed on the vermiculite in each container at a minimum distance of 1.5 cm from the plant. **(A)** Time course of colonization of *R. japonicum*. Numbers indicate days post-inoculation (dpi). Damage caused by SR1 to **(B)** *Physcomitrella patens* (6 dpi), **(C)** *Hypnum plumaeforme* (4 dpi), and **(D)** *Marchantia polymorpha* (4 dpi). Bars = 1 cm.

Colonization of mosses, liverwort and seed plant species by SR1 was observed in plants grown on vermiculite in Petri dishes or Plant Boxes. The hyphae of pure cultures of SR1 placed on vermiculite without plants grew relatively slowly, i.e., 2–3 mm during the 7-day culture period. In contrast, SR1 hyphal growth was visible at 2 days post-inoculation (dpi) when incubated with non-vascular plants, and plants that the hyphae contacted had withered by 4–6 dpi (Figure 4). All mosses and liverworts inoculated, namely *R. japonicum*, *P. patens*, *H. plumaeforme*, and *M. polymorpha*, were killed by SR1.

**FIGURE 5:**
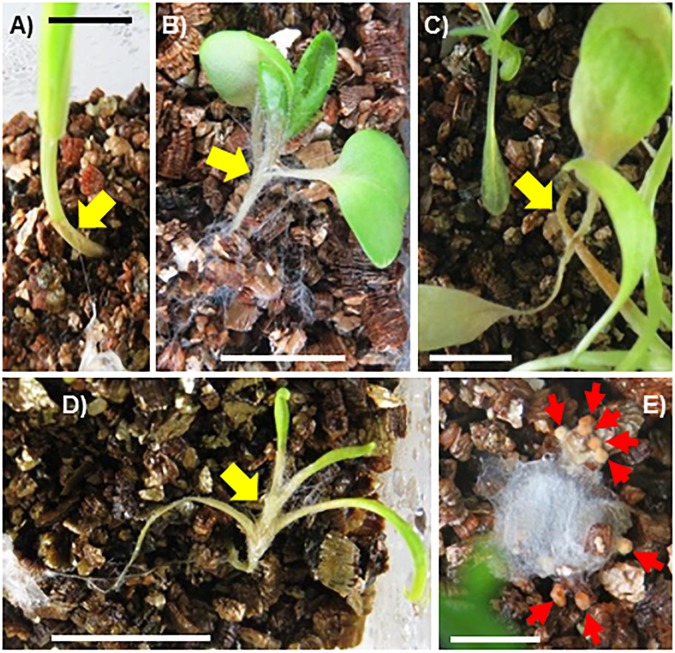
Fungal isolate SR1 is a multihost pathogen of seed plants. **(A)** *Hordeum vulgare* (18 dpi), **(B)** *Brassica rapa* var. *pekinensis* (8 dpi), **(C)** *Lactuca* *sativa* (15 dpi), and **(D)** *Spinacia oleracea* (14 dpi). Yellow arrows indicate areas where hyphae are growing and plants are browning and/or withering. Each bar = 1 cm. **(E)** Sclerotia (red arrows) observed on inoculant applied to *S. oleracea* (14 dpi). Note that growth of hyphae is limited. Bar = 5 mm.

**FIGURE 6:**
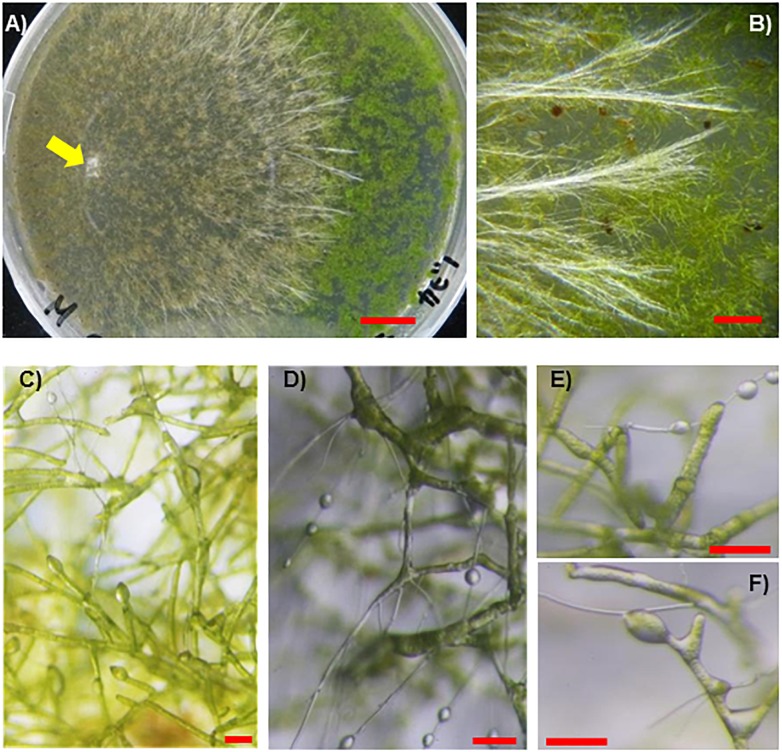
Fungal isolate SR1 directly infects protonemata of *P. patens*. **(A)** Development of lesions infected by SR1 on protonemata of *P. patens* (4 dpi). Yellow arrow indicates inoculant. **(B)** Typical bundles of hyphae on protonemata (4 dpi). **(C)** Micrograph of protonema cells at an early stage of colonization (1 dpi). Note that several cells are slightly swollen, and droplets of secretory fluid can be observed. **(D–F)** SR1-infected protonema at 4 dpi. Note that the cells are slightly swollen, and droplets can be observed. Red bars = 1 cm **(A)**, 1 mm **(B)**, 100 μm **(C–F)**.

When incubated with vascular plant seedlings, the SR1 hyphae grew and reached the plants at 8–11 dpi (Figure 5), except for cultures with plants of *S. oleracea* in which the hyphae grew more slowly. The plant tissues colonized by the hyphae turned brown or white and withering was observed at 11–18 dpi (Figure 5A–D). Unlike the other species, the majority of the hyphae (over 90%) formed sclerotia in the boxes containing *S. oleracea*, although hyphal growth was minimal (Figure 5E). However, hyphae grew from several pieces of inoculants and, once the hyphae reached *S. oleracea* plants (less than 10% of inoculants), the plant was covered by the hyphae, became discolored, and wilted within 14 dpi (Figure 5D). The hyphae of SR1 grew in the plant tissues (Supplementary Figure S2) and developed sclerotia. These results indicated that SR1 is a multihost colonizer of bryophytes, liverworts, and seed plants. SR1 also quickly grew on protonemata of *P. patens* (Figure 6A,B). The protonema cells colonized by hyphae were swollen, and droplets of fluid developed on the hyphae (Figure 6C–F). The droplets of secretory fluid were also observed on the pure-cultured hyphae on the agar plate, especially during development of sclerotia, 1 week after inoculation. Subsequently, the droplets gradually disappeared, except those around the sclerotia (see also Figure 2A).

### SR1 Secretory Fluid Induces ROS

**FIGURE 7:**
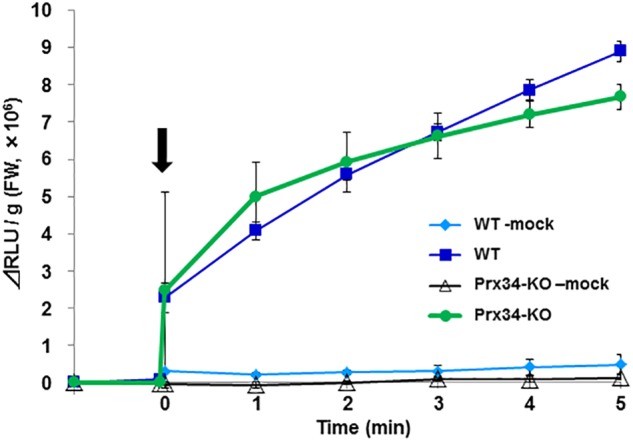
Reactive oxygen species generation by *P. patens* protonema in response to secretory fluid from fungal isolate SR1. Reactive oxygen species was measured by MCLA chemiluminescence intensity. The arrow indicates the time point of fluid application. WT, wild type; KO, Prx34-KO. Mock indicates water-treated control. Error bars indicate the standard deviation (*n* = 3).

It was difficult to maintain *R. japonicum* gametophytes under the aseptic culture condition, therefore further analysis was performed using *P. patens* as a model species. We collected the secretion fluid from SR1 hyphae and examined its effect on ROS generation by *P. patens* protonemata. The fungal secretion rapidly induced ROS production (Figure 7). Production of ROS can be induced also by chitin or chitosan, which involves the peroxidase Prx34 in *P. patens* (Lehtonen et al., 2009), and the chitosan-treated Prx34-KO mutant generates only weak ROS signals, as detected by MCLA chemiluminescence (Lehtonen et al., 2012a). In response to application of the SR1 secretory fluid, similar amounts of ROS were detected in wild-type (WT) *P. patens* and the Prx34-KO mutant (Figure 7).

### Moss Peroxidase Partially Protects *P. patens* Against SR1

**FIGURE 8:**
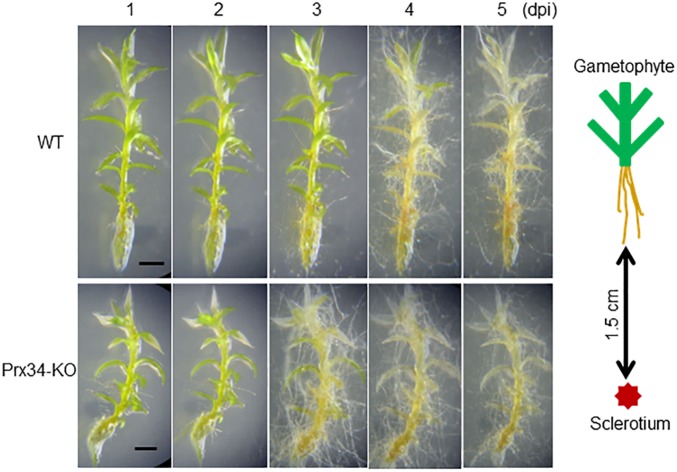
Time course of hyphal growth of fungal isolate SR1 on the gametophyte of the wild type (WT; upper half) and Prx34-KO mutant (lower half) of *P. patens*. A schematic illustration of the experimental design is shown to the right of the images. Sixteen gametophytes of WT were tested and the results were consistent. For the Prx34-KO mutant, 15 or 16 gametophytes from each of three Prx34-KO lines were tested and the same results were observed. A representative result is shown. Each bar = 1 mm.

*Physcomitrella patens* was inoculated by placement of pure-cultured sclerotia of SR1 at a distance of 1.5 cm from gametophytes of the Prx34-KO mutant and the WT. Hyphal growth was quickly initiated, with hyphae reaching the gametophytes at 3 dpi and quickly covering the plants (Figure 8). The WT plants were totally covered by hyphae by 4–5 dpi. In contrast, the hyphae showed more rapid growth on the Prx34-KO mutant and the gametophytes were totally covered by hyphae by 3–4 dpi.

### A Bacterium Antagonistic to Fungal Isolate SR1 Coexists With *R. japonicum*

**FIGURE 9:**
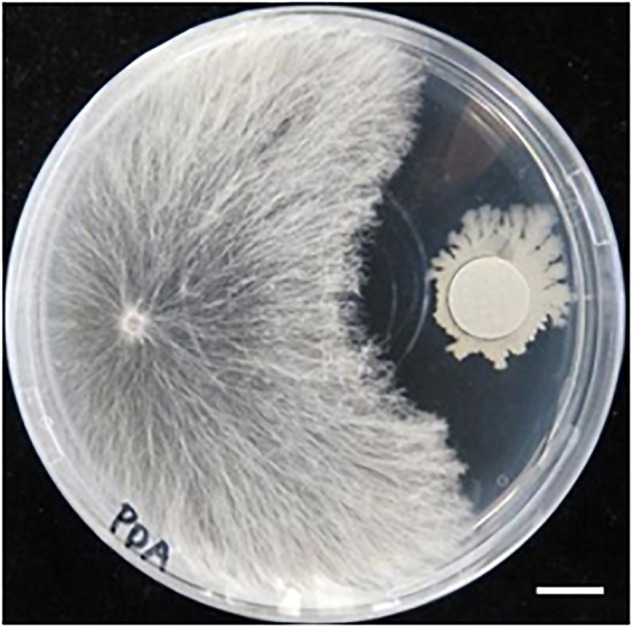
Antagonistic activity of bacterium isolate R-1 against fungal isolate SR1. Halo formation is shown. A hyphal tip of SR1 was inoculated on the left of the PDA plate, whereas R-1 was inoculated with filter paper (diameter 1.2 cm) pre-soaked in the bacterial culture. The plate was cultured at 28°C in the dark. Bar = 1 cm.

**FIGURE 10:**
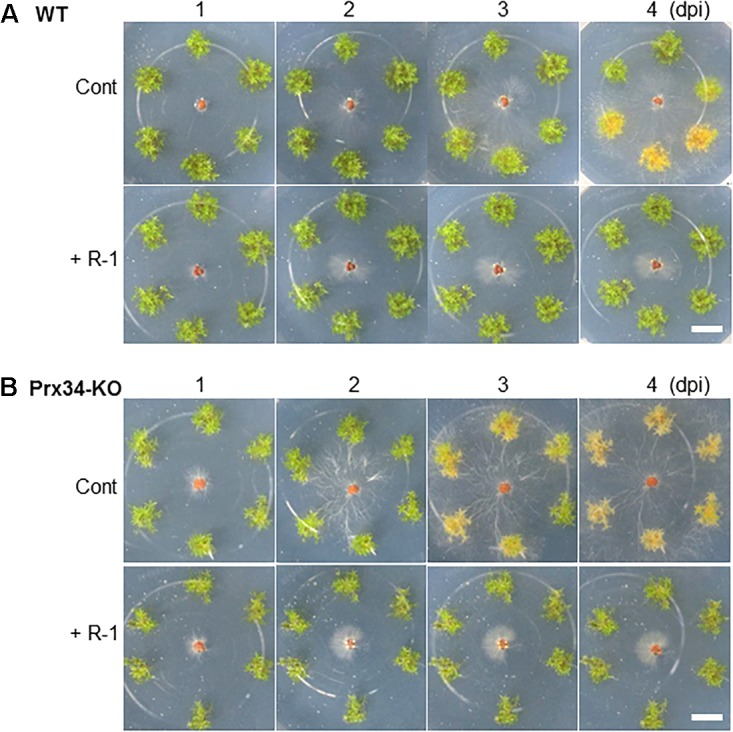
Effect of pre-inoculation with bacterium isolate R-1 on protection of *P. patens* wild type (upper half, **A**) and the Prx34-KO mutant (lower half, **B**) against fungal isolate SR1. Time course of hyphal growth and browning of moss colonies is shown. The bacterium R-1 was inoculated onto the moss 1 week prior to SR1 inoculation. Note that no influence was observed with R-1 inoculation only (applicable for each control). A representative result from five repetitions is shown. Bars = 1 cm.

We previously isolated a bacterium R-1, possibly *B. amyloliquefaciens*, that shows antagonistic activity against fungi (Supplementary Figure S3). The bacterium R-1 showed antagonistic activity against the fungal isolate SR1 (Figure 9), but inoculation with R-1 showed no influence on *P. patens* gametophytes (see also Figure 10). When sclerotia were inoculated at 2 cm distance from the colony of the gametophytes, hyphae quickly reached the gametophytes and caused browning on WT plants at 4 dpi (Figure 10), whereas for gametophytes pre-inoculated with R-1, growth of hyphae was distinctly repressed and new sclerotia quickly developed on the inoculated sclerotia. Gametophytes of the Prx34-KO mutant were more quickly damaged by SR1 compared with WT gametophytes, as described above, whereas Prx34-KO gametophytes pre-inoculated with R-1 showed no symptoms and the fungus quickly developed new sclerotia (Figure 10).

## Discussion

Knowledge of the microbial flora associated with mosses is expected to be important for control and preservation of global and local environments. Moss–fungus interactions have been extensively studied from ecological and biological perspectives (e.g., Shcherbakov et al., 2013; Szentes et al., 2013). Fungal communities and biomass in the bryophyte communities in different seasons have also been studied (Davey et al., 2012). Many mosses are economically important, because they can be used for greening, gardening and as ornamentals. However, the contributions of microbes to moss quality are often poorly understood. In addition, given that mosses may be alternative hosts for plant-pathogenic fungi (Lehtonen et al., 2012b; Ueta and Tojo, 2016), information on moss-associated fungi is important for pest control in horticulture and agriculture.

In the present study, we isolated a fungus that causes devastating damage to Sunagoke moss (*R. japonicum*), which is used for greening of buildings. ITS sequence data indicated that the SR1 isolate was a strain of *S. delphinii* which, to the best of our knowledge, has not been previously recognized as a pathogen of *R. japonicum*. However, Morita (2009, 2013) reported that *R. japonicum* is infected by *S. rolfsii*, a fungus reported to be a pathogen of several bryophytes. Okabe (2002) proposed that *S. delphinii* and *S*. *rolfsii* are conspecific. Strain SR1 of *S. delphinii* described in the current study was shown to have a wide host range, including a liverwort, mosses, and several mono- and dicotyledonous seed plants, but colonization progressed more slowly in seed plants compared with non-vascular plants. We also observed that most *S. oleracea* plants escaped from colonization because the hyphae grew poorly, if at all. Unidentified factor(s) of plants may stimulate chemotaxis of fungi and *S. oleracea* may produce only limited amounts of such factor(s), or alternatively *S. oleracea* may produce a factor(s) that disturbs the chemotaxis.

*Sclerotium* spp. belong to a group of oxalate-secreting fungi. SR1 secretes an oxalic acid containing fluid (Supplementary Figure S4). The present results indicated that bryophytes may be sensitive to oxalates and, in this respect, resemble seed plants. Oxalate is suggested to be associated with development of symptoms through induction of ROS. We detected ROS using MCLA, which is a chemiluminescent dye to detect ROS but generate weak signals against H_2_O_2_ (Kambayashi and Keiki, 2003), therefore the present results suggest that ROS may be produced during oxalate decomposition by oxalate oxidase (Opaleye et al., 2006). Production of ROS is also induced by chitin in *P. patens*, which involves activity of the peroxidase Prx34. The WT and the Prx34-KO mutant also showed ROS generation in response to treatment with the SR1 fungal secretion, which suggested that ROS induction by the secretion is independent of Prx34 peroxidase activity. This result is consistent with the inhibitory effect of oxalate on peroxidase (Zhang and Shao, 2015), and with the possibility that the fungal secretion includes oxalate and ROS are generated through oxalate metabolism, which may be part of the moss response to the secretion. *P. patens* is suggested to have two putative oxalate oxidases, XP_001776467.1 and BAD86497.1^2^, and 10 germin-like proteins are expressed (Nakata et al., 2004). However, no information is available on the catalytic activity of oxalate oxidase in *P. patens*. Tolerance to oxalate-producing fungi can be enhanced by introduction of oxalate metabolism to seed plants (Kesarwani et al., 2000), which suggests that it may be possible to breed a strain of *R. japonicum* that shows high tolerance to *Sclerotium* spp. by the same strategy. We observed that secretion of the putative oxalate-containing exudate seemed to be enhanced during colonization. The mechanism by which the moss induces the fungal secretion is an interesting topic for future investigation.

We observed that fungal colonization of *P. patens* can be prevented by two factors, namely a peroxidase expressed by the moss and an antagonistic bacterium that coexists in the moss habitat. Host-plant peroxidases may influence fungal colonization. We previously reported that Prx34, a peroxidase of *P. patens*, plays an important role in protection of the moss against fungal colonization and that the Prx34-KO mutant is more susceptible to fungal attack than wild-type *P. patens* (Lehtonen et al., 2009). Involvement of Prx34 in protection against fungi was further demonstrated in the present study, but the effect was insufficient to prevent SR1. The secreted peroxidase itself and/or the products of its catalytic activity, including ROS, may partially suppress hyphal growth. Involvement of peroxidase in recognition of pathogens and protection of host plants has been well studied (Bindschedler et al., 2006; Daudi et al., 2012; O’Brien et al., 2012; Camejo et al., 2016). Many studies have raised the possibility of enhancing resistance against pathogens by introduction of recombinant peroxidases (Wally and Punja, 2010). Given that Prx34 is secreted constitutively into the culture medium, whereas the secretion is significantly stimulated by chitosan (Lehtonen et al., 2014), Prx34 itself or products of its activity may partially affect hyphal growth. Accordingly, the hyphal growth rate was higher in the Prx34-KO mutant than in WT *P. patens*. When hyphae come into contact with moss cells, peroxidase may be rapidly secreted, albeit not in sufficient quantity to protect the moss, or the peroxidase may be inhibited by the fungal exudates. In contrast, the bacterium isolate R-1 (a putative strain of *B. amyloliquefaciens*) can prevent growth of certain fungi, including *S. delphinii* and *Fusarium avenaceum* (Akita et al., 2011) (Supplementary Figure S3), both of which cause diseases in *R. japonicum*. The mechanism by which R-1 influences SR1 growth requires further study, but *B. amyloliquefaciens* is known to produce several antifungal agents, such as iturin A (Yu et al., 2002). Given that R-1 showed no negative effect on growth of *R. japonicum* (Supplementary Figure S5), application of R-1 is promising as a novel protective treatment for the moss. One of the important advantages of greening with *R. japonicum* is the potential for long-term maintenance-free use. Therefore, management of the moss-associated bacteria may be vital for successful moss greening. Moss colonies are promising sources of bacteria that are capable of controlling pathogens (e.g., Aleti et al., 2016). Thus, it will be necessary to study the roles of R-1 and other beneficial bacteria in maintaining high moss quality in panels prepared for greening.

## Author Contributions

MaT and MiT made experimental design and carried out laboratory experiments. MA and JV interpreted the data and wrote the manuscript.

## Conflict of Interest Statement

The authors declare that the research was conducted in the absence of any commercial or financial relationships that could be construed as a potential conflict of interest.

## Footnotes

1 http://biology4.wustl.edu/moss/Mediarecipes.pdf

2 http://www.cosmoss.org

## Supplementary Material

FIGURE S1 An example of greening of a roof with *Racomitrium japonicum*.

FIGURE S2 Fungal isolate SR1 hyphae growing on an infected host plant. Leaf tissue of *Hordeum vulgare* is shown as an example. The hyphae were stained with Cotton blue. Bar = 10 μm.

FIGURE S3 Halo formation by bacterium isolate R-1, a putative strain of *Bacillus amyloliquefaciens*, against *Fusarium avenaceum* on malt agar medium. Bar = 1 cm.

FIGURE S4 SR1 secretes oxalate containing fluid. Relationship between culture period and the oxalic acid content of secretion fluid was shown **(A)**. Secretion fluid was collected from each sclerotium by careful pipetting from the culture plate (see Figure 2). The fluid from one plate was mixed together and the oxalic acid content was measured by a commercial kit (Enzykit Oxalic acid, J.K.international, Tokyo, Japan) according to the instruction manual. The oxalic acid content was independent with the culture period. The content was varied but significantly higher than the content of oxalic acid in the medium of which *P. patens* started browning (**B**, 3 days cultured *P. patens* in 0.5 mM oxalic acid containing 1/10 BCD liquid medium).

FIGURE S5 Effect of bacterium isolate R-1 on growth of *Racomitrium japonicum*. The bacterium was cultured overnight using LB liquid medium in an Erlenmeyer flask at 100 rpm at 37°C. The culture was centrifuged at ca. 1400 ×*g* at 4°C for 3 min. The supernatant was discarded and the pellet was rinsed with sterilized water three times, then the pellet was resuspended in sterilized water and adjusted to OD_600_ = 3.5. Clean gametophytes of *R. japonicum* were soaked in the bacterial suspension for 3 min and then transplanted to an autoclaved Plant Box containing 95 g vermiculite saturated with BCD medium. Plants were cultured under continuous illumination (ca. 80 μmol m^-2^ s^-1^) at 23°C for 3 weeks. Error bars indicate the standard deviation (*n* = 38 for the non-inoculated control and *n* = 40 for the R-1 inoculation treatment).
