# Supplementary figures and images for: Sunagoke Moss (Racomitrium japonicum) Used for Greening Roofs Is Severely Damaged by Sclerotium delphinii and Protected by a Putative Bacillus amyloliquefaciens Isolate
Source: Front Microbiol. 2019 Feb 28;10:372. doi: 10.3389/fmicb.2019.00372 (PMC6403164; doi:10.3389/fmicb.2019.00372)

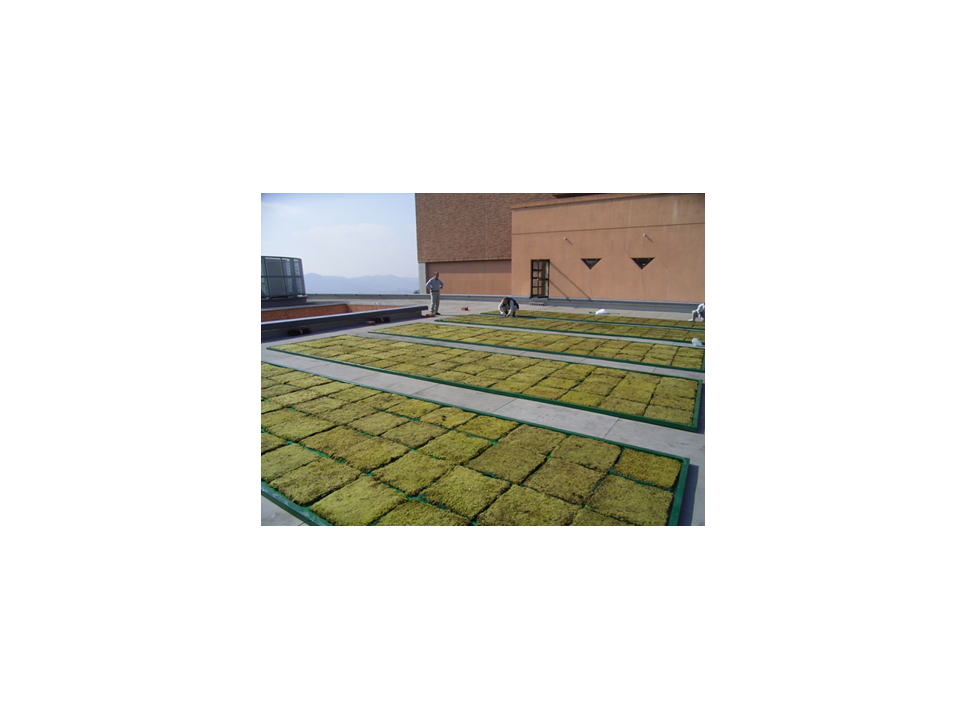

Supplement: FIGURE S1 — An example of greening of a roof with Racomitrium japonicum. [file Image_1.TIF]

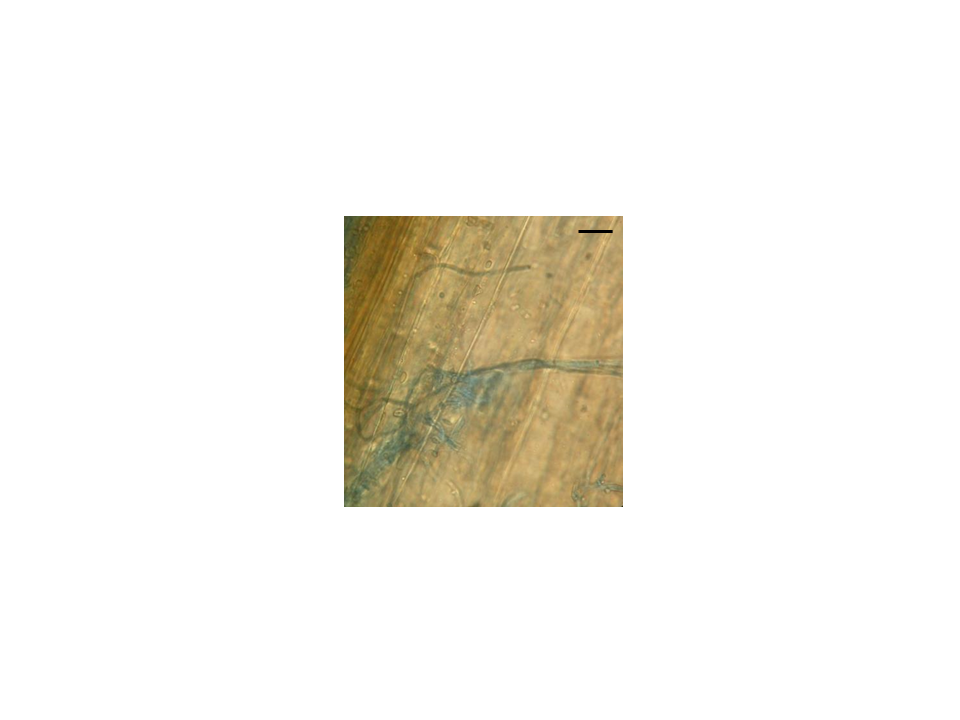

Supplement: FIGURE S2 — Fungal isolate SR1 hyphae growing on an infected host plant. Leaf tissue of Hordeum vulgare is shown as an example. The hyphae were stained with Cotton blue. Bar = 10 μm. [file Image_2.TIF]

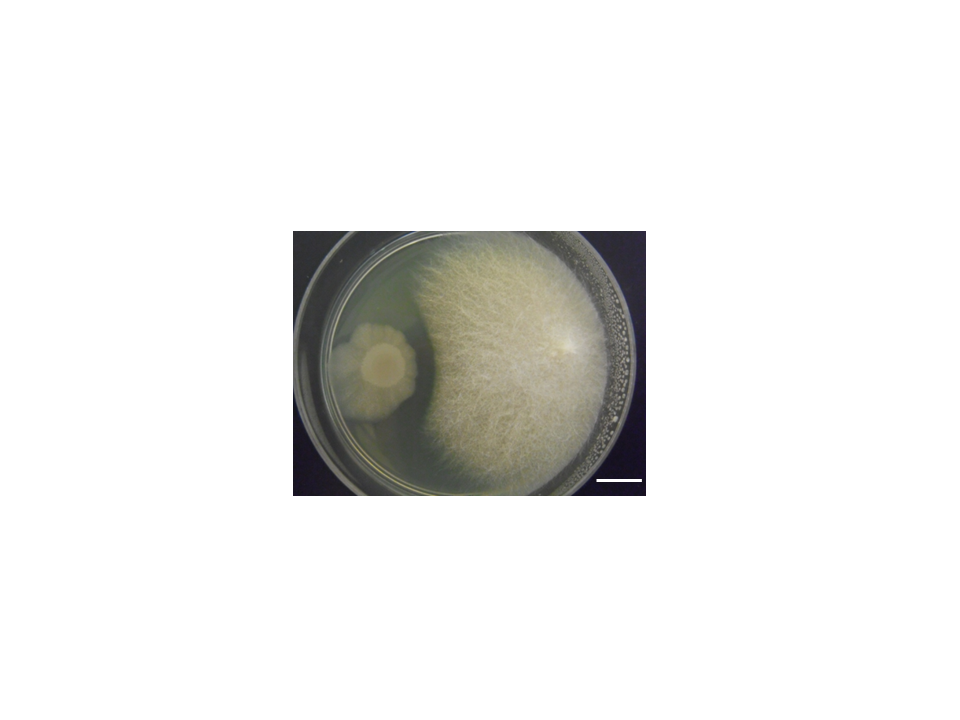

Supplement: FIGURE S3 — Halo formation by bacterium isolate R-1, a putative strain of Bacillus amyloliquefaciens, against Fusarium avenaceum on malt agar medium. Bar = 1 cm. [file Image_3.TIF]

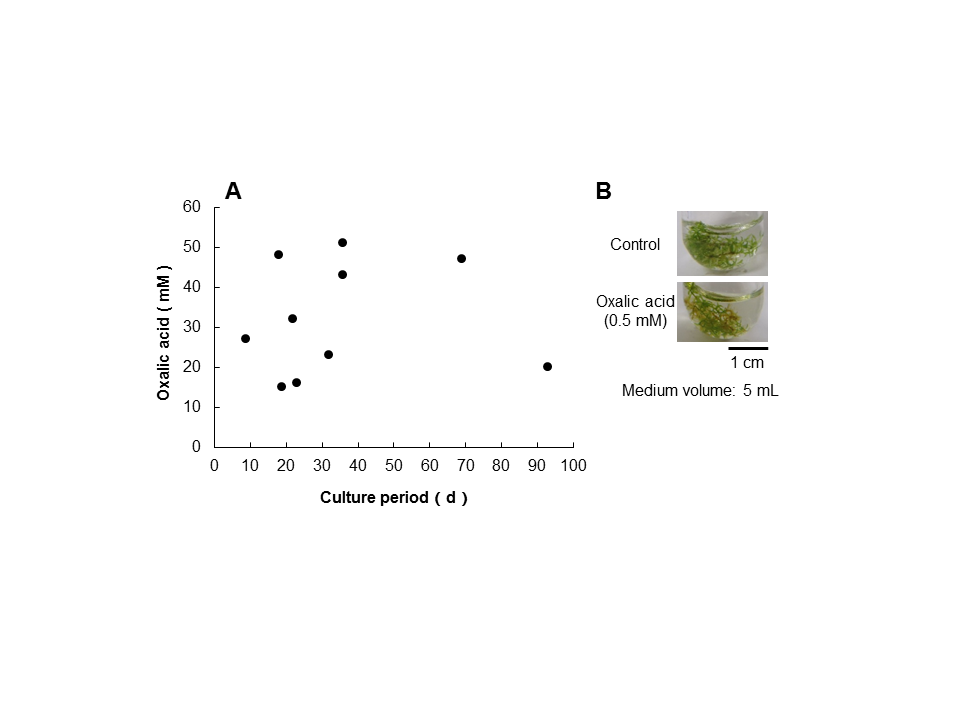

Supplement: FIGURE S4 — SR1 secretes oxalate containing fluid. Relationship between culture period and the oxalic acid content of secretion fluid was shown (A). Secretion fluid was collected from each sclerotium by careful pipetting from the culture plate (see Figure 2). The fluid from one plate was mixed together and the oxalic acid content was measured by a commercial kit (Enzykit Oxalic acid, J.K.international, Tokyo, Japan) according to the instruction manual. The oxalic acid content was independent with the culture period. The content was varied but significantly higher than the content of oxalic acid in the medium of which P. patens started browning (B, 3 days cultured P. patens in 0.5 mM oxalic acid containing 1/10 BCD liquid medium). [file Image_4.TIF]

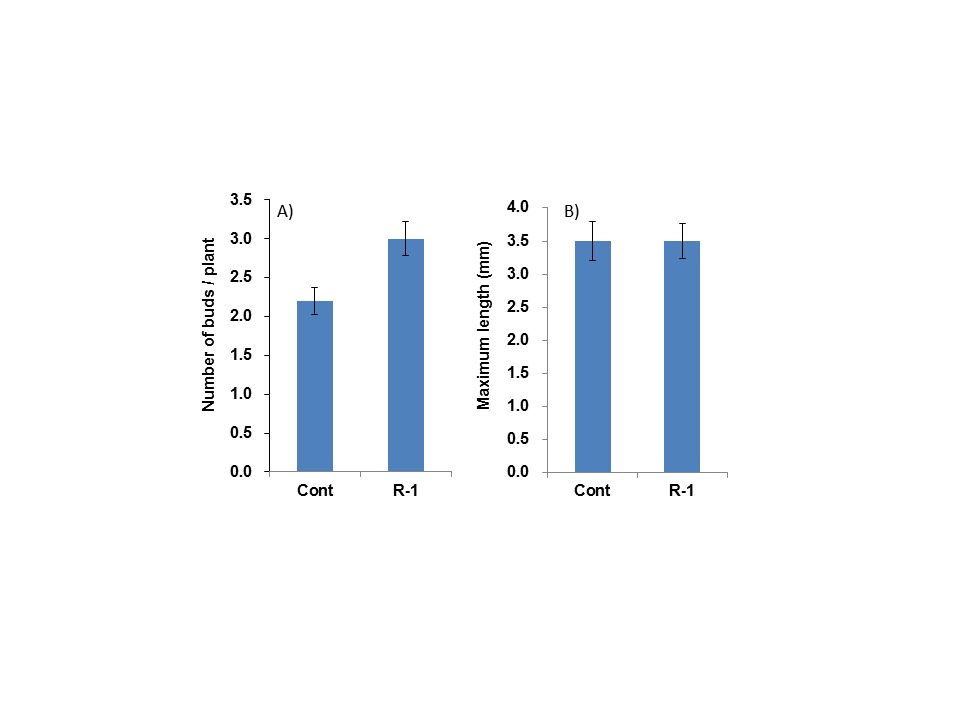

Supplement: FIGURE S5 — Effect of bacterium isolate R-1 on growth of Racomitrium japonicum. The bacterium was cultured overnight using LB liquid medium in an Erlenmeyer flask at 100 rpm at 37°C. The culture was centrifuged at ca. 1400 ×g at 4°C for 3 min. The supernatant was discarded and the pellet was rinsed with sterilized water three times, then the pellet was resuspended in sterilized water and adjusted to OD600 = 3.5. Clean gametophytes of R. japonicum were soaked in the bacterial suspension for 3 min and then transplanted to an autoclaved Plant Box containing 95 g vermiculite saturated with BCD medium. Plants were cultured under continuous illumination (ca. 80 μmol m-2 s-1) at 23°C for 3 weeks. Error bars indicate the standard deviation (n = 38 for the non-inoculated control and n = 40 for the R-1 inoculation treatment). [file Image_5.TIF]
